# Diabetes increases the risk of heart failure in myocarditis: a propensity-matched nationwide database analysis

**DOI:** 10.1093/eschf/xvag064

**Published:** 2026-02-23

**Authors:** Rayane El-Khoury, Shadi Mahmoud, Soha Dargham, Ziyad Mahfoud, Amin Jayyousi, Jassim Al Suwaidi, Charbel Abi Khalil

**Affiliations:** Department of Genetic Medicine, Weill Cornell Medicine-Qatar, Doha, Qatar; Department of Genetic Medicine, Weill Cornell Medicine-Qatar, Doha, Qatar; Department of Medical Education, Weill Cornell Medicine-Qatar, Doha, Qatar; Biostatistics Core, Weill Cornell Medicine-Qatar, Doha, Qatar; Department of Endocrinology, Hamad Medical Corporation, Doha, Qatar; Heart Hospital, Hamad Medical Corporation, Doha 3050, Qatar; Department of Genetic Medicine, Weill Cornell Medicine-Qatar, Doha, Qatar; Heart Hospital, Hamad Medical Corporation, Doha 3050, Qatar; Joan and Sanford I, Weill Department of Medicine, Weill Cornell Medicine, New York, NY, USA; Department of Population Health Sciences, Weill Cornell Medicine, New York, NY, USA

**Keywords:** Myocarditis, Diabetes, National readmission database, Cardiovascular disease, Cardiology

## Abstract

**Background and Aims:**

The impact of diabetes on non-atherosclerotic cardiac disease has not been studied extensively. We aimed to assess the in-hospital and long-term effects of diabetes in patients hospitalized for myocarditis.

**Methods:**

The Nationwide Readmissions Database (2016–2020) was used to identify adults hospitalized with a primary diagnosis of myocarditis. Patients were stratified by the presence of diabetes, and those discharged alive were followed for a calendar year. The primary outcome was in-hospital mortality. Secondary outcomes included in-hospital ventricular fibrillation, ventricular tachycardia, acute renal failure, cardiogenic shock, heart failure, and one-year all-cause readmission, readmission for heart failure, and mortality. Multivariable logistic and Cox regression models were applied, and propensity score matching was performed as a sensitivity analysis.

**Results:**

Among 8826 adults with myocarditis, 951 (11%) had diabetes. Compared with patients without diabetes, those with diabetes were older, had a higher prevalence of comorbidities, and showed an increased adjusted risk of in-hospital acute renal failure [aOR = 1.74 (95% CI: 1.42–2.12)], heart failure [aOR = 1.62 (95% CI: 1.37–1.91)], cardiogenic shock [aOR = 1.36 (95% CI: 1.04–1.78)], but not of mortality, ventricular fibrillation, and ventricular tachycardia. In one year, diabetes was not associated with higher adjusted risks of all-cause readmission or mortality [aHR = 0.81 (95% CI: 0.41–1.60) and aHR = 0.81 (95% CI: 0.68–0.97), respectively]. However, it was associated with a higher risk of readmission for heart failure [aHR = 1.16 (95% CI: 1.02–1.31)]. These associations remained consistent in propensity score-matched analyses.

**Conclusion:**

Diabetes independently increases the risk of in-hospital and one-year heart failure in patients with myocarditis.

## Graphical Abstract

**Graphical Abstract:**
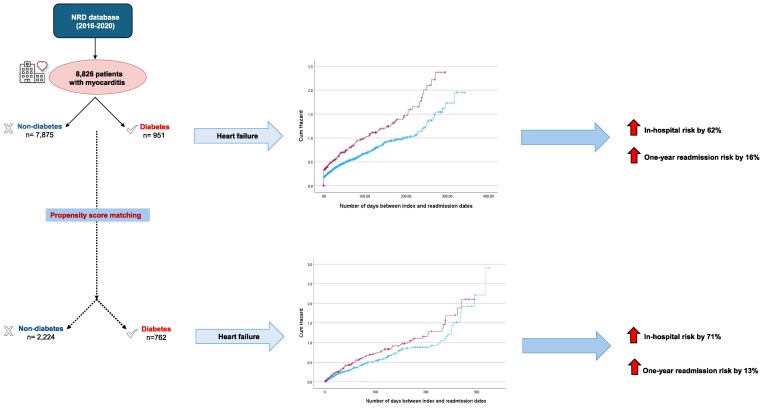

## Introduction

Myocarditis is characterized by inflammation of the myocardium, caused by various infectious and non-infectious triggers.^1,2^ It represents a substantial global health burden, with nearly 2 million cases diagnosed annually.^3^ In 2019, a total of 340 349 deaths due to myocarditis were reported worldwide.^4^ Myocarditis significantly increases the risk of sudden cardiac death (SCD),^5^ accounting for approximately 10 to 20% of all cases in young adults, and is the third most common cause of SCD in this age group.^6–9^

Diabetes is a major public health issue.^10^ In 2024, the World Health Organization (WHO) reported that more than 800 million adults were living with diabetes, representing a nearly fourfold increase since 1990.^11^ Diabetes is associated with endothelial dysfunction and an increased risk of cardiovascular disease.^12,13^ Atherosclerotic cardiovascular disease (ASCVD) is the primary cause of morbidity and mortality in individuals with diabetes.^13^ Studies consistently show a 2 to 4-fold increased risk of coronary artery disease (CAD), peripheral artery disease, and stroke in patients with diabetes compared to those without, accounting for approximately 80% of deaths in this population.^14^ Diabetes is also associated with a higher risk of heart failure (HF).^15^ It is estimated that 20 to 40% of HF patients have diabetes, which is associated with a worse prognosis.^16^ Although the most common aetiology of HF in patients with diabetes is ischaemic heart disease, recent studies have shown a high prevalence of diabetic cardiomyopathy (DCM),^17^ a distinct diabetes complication characterized by structural and functional changes in the myocardium, irrespective of the presence of ASCVD .^18^

Diabetes and myocarditis share several, often overlapping, pathophysiological mechanisms centred on inflammation, immune dysregulation, and metabolic stress, leading to myocardial damage. Hence, we sought to examine the impact of diabetes on clinical outcomes of patients hospitalized for myocarditis.

## Methods

### Data source

The study utilized patient data from the Nationwide Readmissions Database (NRD), a component of the Healthcare Cost and Utilization Project (HCUP).^19^ The NRD collects de-identified patient data from approximately 18 million discharges annually, as well as subsequent readmissions.^20^ Data include clinical characteristics and outcomes, entered into the database using relevant codes from the International Classification of Diseases (ICD). Access to the NRD database required a data use agreement between HCUP and Weill Cornell Medicine-Qatar. Our study adhered to local and international ethical requirements and obtained administrative IRB approval (record number 21-0001); therefore, a consent form was not required. The study follows the Strengthening the Reporting of Observational Studies in Epidemiology (STROBE) guidelines (Supplementary Table S1).

### Patients and outcomes

Patients were included in this analysis if they had a primary diagnosis of myocarditis (ICD-10 codes: I40.0, I40.1, I40.8, I40.9, and I51.4). Patients younger than 18 years of age were excluded. Patients were then stratified into two groups based on the presence or absence of a secondary diagnosis of diabetes (ICD-10 codes: E08x, E09x, E10x, E11x, E13x, O24.1x, O24.3x, O24.8, and O24.9). The primary outcome was in-hospital mortality. Secondary outcomes were in-hospital ventricular fibrillation (VFIB), acute renal failure (ARF), cardiogenic shock, heart failure (HF), ventricular tachycardia (VTAC), as well as one-year outcomes. One-year secondary outcomes were defined as all-cause readmissions, readmission for heart failure, and mortality occurring within the calendar year following the index hospitalization. All comorbidities, aetiologies, and outcomes were defined using ICD-10 codes (Supplementary Table S2) and analysed as dichotomous variables. Patients were tracked using their unique NRD identifiers *(NRD_VisitLink)* to capture hospitalizations and readmissions. If patients were readmitted, they were classified as all-cause or heart failure-related based on their ICD-10 codes. All-cause readmissions were defined as any inpatient readmission for cardiovascular and non-cardiovascular causes, other than heart failure, regardless of the underlying diagnosis. One-year mortality was defined as death occurring in-hospital within one year of discharge, among patients who were readmitted alive. The NRD does not capture out-of-hospital mortality, so deaths were only identified if they occurred during an inpatient hospitalization.

### Statistical analysis

#### Baseline characteristics

After merging all data from 2016 to 2020 and excluding patients with missing data, diabetes patients admitted for myocarditis were compared to their non-diabetes counterparts. Baseline characteristics were compared using an independent *t*-test or a Pearson Chi-square test, with data presented as mean (SD) or number (%).

#### Multivariable logistic regression

Pearson’s Chi-square test was also used to generate in-hospital Odds ratios (OR) along with their 95% confidence interval (CI). Non-diabetes patients were used as the reference category. Variables with *P*-values < .05 in the univariate comparisons between diabetes and non-diabetes groups were eligible for inclusion into the multivariable analysis. Multivariable logistic regression was conducted to account for baseline differences and identify independent predictors of adverse in-hospital outcomes. The model included age, gender, obesity, smoking, hypertension, dyslipidaemia, peripheral vascular disease (PVD), valvular heart disease (VHD), chronic kidney disease (CKD), CAD, and cerebrovascular disease. The results of the adjusted analyses were reported in terms of adjusted ORs (aORs) along with their 95% CI.

#### Time-to-event analysis

Kaplan–Meier (KM) curves were generated to visualize time-to-event data and compare the groups’ cumulative 1-year mortality and heart failure risk. We used a log-rank test to compare outcomes and a Cox proportional hazard model to adjust for different baseline characteristics on admission. Values are presented as hazard ratios (HR) with their respective 95% CI. The significance threshold for all analyses was set at a *P*-value of ≤ .05.

#### Sensitivity analysis

A propensity score matching was performed as a sensitivity analysis. The propensity scores were matched in a 1:3 ratio with exact matching on age (categorical), gender, smoking, hypertension, dyslipidaemia, and CAD. The comparison between diabetes and non-diabetes was adjusted for the variables that were significant in the original analysis. Analyses were conducted in SPSS Statistics version 29.0 (IBM Corp.).

## Results

### Study population

**Figure 1:**
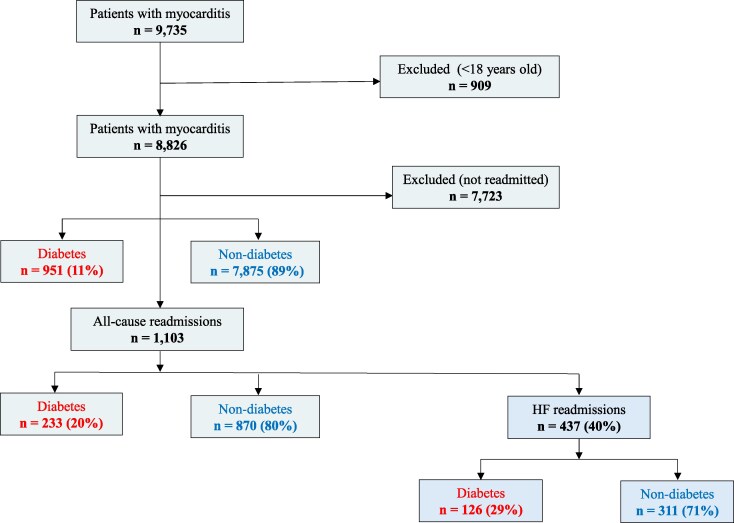
Flowchart showing patient selection and stratification

A total of 9735 patients were hospitalized for myocarditis between 2016 and 2020 (*Figure 1*). After excluding 909 patients younger than 18 years, 8826 adults with myocarditis were included in the analysis. Of those included, 951 (11%) had diabetes. Among these 8826 patients, 8620 were discharged alive, while the remaining 206 patients died during hospitalization and were therefore not included in the readmission analysis. Among the 8620 patients, there were 1103 all-cause readmissions within one year; 223 (20%) of whom had diabetes. Among all-cause readmissions, 437 (40%) were due to heart failure, including 126 cases (29%) with diabetes and 311 (71%) without diabetes.

### Baseline characteristics

*My*ocarditis patients with diabetes were, on average, 15 years older than their non-diabetes counterparts (56[16] vs. 41[18], respectively, *P* < .001) (*Table 1*). Over 75% of non-diabetes patients were younger than 55 years of age, whereas only 42% of diabetes patients fell within the same age range (*P* < .001 for the comparison across all age ranges). There were more males than females among all patients. As expected, all cardiovascular risk factors were significantly more prevalent among patients with diabetes. The proportion of patients with PVD, VHD, CKD, and CAD was higher in patients with diabetes (*P* < .001 for all). Unspecified myocarditis, comprising interstitial myocarditis and myocardial fibrosis, was the most common aetiology in all patients, followed by infective myocarditis (viral or bacterial). No statistically significant difference in either aetiology was observed between the groups. There was no statistical difference in the remaining aetiologies (isolated autoimmune and unspecified myocarditis).

**Table 1:** Comparison of baseline demographics between myocarditis patients

	Diabetes *n* = 951	Non-diabetes *n* = 7875	*P*-value^a^
	*n* (%)	*n* (%)	
Age			
Mean (SD)	56 (16)	41 (18)	<.001
<55	402 (42.3)	6068 (77.1)	<.001
55–64	210 (22.1)	850 (10.8)	
65–74	214 (22.5)	601 (7.6)	
75–84	106 (11.2)	273 (3.5)	
>84	19 (2.0)	83 (1.1)	
Gender			
Male	500 (52.6)	5158 (65.5)	<.001
Female	451 (47.4)	2717 (34.5)	
Comorbidities			
Obesity	322 (33.9)	1145 (14.5)	<.001
Smoking	190 (20.0)	1134 (14.4)	<.001
Hypertension	739 (77.7)	2278 (28.9)	<.001
Dyslipidaemia	535 (56.3)	1563 (19.8)	<.001
PVD	48 (5.0)	189 (2.4)	<.001
VHD	128 (13.5)	642 (8.2)	<.001
CKD	172 (18.1)	277 (3.5)	<.001
CAD	331 (34.8)	1210 (15.4)	<.001
Cerebrovascular disease	51 (5.4)	137 (1.7)	<.001
Etiology			
Infective acute myocarditis^b^	314 (33.0)	2522 (32.0)	.536
Isolated acute myocarditis^c^	34 (3.6)	292 (3.7)	.838
Other and unspecified acute myocarditis^d^	212 (22.3)	1613 (20.5)	.193
Unspecified myocarditis^e^	391 (41.1)	3448 (43.8)	.117

CAD, Coronary Artery Disease; CKD, Chronic Kidney Disease; PVD, Peripheral Vascular Disease; VHD, Valvular Heart Disease.

^a^Covariates with a *P*-value ≤.05 were considered statistically significant.

^b^Myocarditis caused by a specific infectious agent, such as a virus or bacterium.

^c^Acute myocarditis occurring without an apparent infection or autoimmune disease

^d^Acute myocarditis that does not fit into the defined categories but has a known cause.

^e^Myocarditis with no specified cause, or additional classification details.

### In-hospital outcomes

Diabetes patients had significantly higher odds of the primary outcome of in-hospital mortality. It also has significantly higher odds of ventricular fibrillation, acute renal failure, cardiogenic shock, heart failure, and ventricular tachycardia (*Table 2*). However, after multivariable adjustment on statistically significant parameters between diabetes and non-diabetes, only ARF [aOR = 1.74 (95% CI: 1.42–2.12)], HF [aOR = 1.62 (95% CI: 1.37–1.91)], and cardiogenic shock [aOR = 1.36 (95% CI: 1.04–1.78)] remained statistically significant. Supplementary Table S3 shows the predictors of outcomes that are significantly different between groups. Age increases the risk of all those events, irrespective of diabetes. Being female was associated with a lower risk of ARF in patients with diabetes [OR = 0.64 (95% CI: 0.46–0.88)] and those without diabetes [OR = 0.66 (95% CI: 0.56–0.79)]. Unsurprisingly, CKD at inclusion increased this risk by almost 4-fold in diabetes patients and 8-fold in non-diabetes patients. Still, hypertension increased the risk only in non-diabetes patients [OR = 1.32 (95% CI: 1.10–1.60)]. VHD significantly increased the risk of cardiogenic shock in all patients [OR = 1.30 (95% CI: 0.84–2.03), OR = 3.41 (95% CI: 2.86–4.07)] for diabetes and non-diabetes, respectively. CKD increased the risk of HF by almost 2-fold in diabetes patients and 3-fold in non-diabetes patients. Paradoxically, dyslipidaemia was associated with a lower risk of in-hospital events in all patients.

**Table 2:** Comparison of in-hospital cardiovascular events between myocarditis patients among the overall and matched populations.

	Overall population				Propensity scores matched population^b^			
	Diabetes *n* (%)	Non-diabetes *n* (%)	Unadjusted OR (95% CI)	Adjusted OR (95% CI)^a^	Diabetes *n* (%)	Non-diabetes *n* (%)	Unadjusted OR (95% CI)	Adjusted OR (95% CI)^a^
**Mortality**								
No	915 (96.2)	7705 (97.9)	Ref	Ref	733 (96.19)	2154 (96.94)	Ref	Ref
Yes	36 (3.8)	166 (2.1)	**1.83 (1.27–2.64)**	1.00 (0.66–1.50)	29 (3.81)	68 (3.06)	1.25 (0.80–1.95)	1.15 (0.72–1.83)
**Ventricular Fibrillation**								
No	800 (84.1)	7027 (89.2)	Ref	Ref	642 (84.25)	1915 (86.11)	Ref	Ref
Yes	151 (15.9)	848 (10.8)	**1.56 (1.30–1.89)**	1.06 (0.86–1.31)	120 (15.75)	309 (13.89)	1.16 (0.92–1.46)	1.07 (0.84–1.36)
**Acute renal failure**								
No	715 (75.2)	7104 (90.2)	Ref	Ref	574 (75.33)	1913 (86.02)	Ref	Ref
Yes	236 (24.8)	771 (9.8)	**3.04 (2.58–3.59)**	**1.74 (1.42–2.12)**	188 (24.67)	311 (13.98)	**2.01 (1.64–2.47)**	**1.70 (1.35–2.13)**
**Cardiogenic shock**								
No	861 (90.5)	7428 (94.3)	Ref	Ref	686 (90.03)	2073 (93.21)	Ref	Ref
Yes	90 (9.5)	447 (5.7)	**1.74 (1.37–2.20)**	**1.36 (1.04–1.78)**	76 (9.97)	151 (6.79)	**1.52 (1.14–2.03)**	**1.43 (1.06–1.94)**
**Heart failure**								
No	569 (59.8)	6366 (80.8)	Ref	Ref	452 (59.32)	1622 (72.93)	Ref	Ref
Yes	382 (40.2)	1509 (19.2)	**2.83 (2.46–3.26)**	**1.62 (1.37–1.91)**	310 (40.68)	602 (27.07)	**1.85 (1.56–2.20)**	**1.71 (1.42–2.06)**
**Ventricular tachycardia**								
No	865 (91.0)	7380 (93.7)	Ref	Ref	691 (90.68)	2060 (92.63)	Ref	Ref
Yes	86 (9.0)	495 (6.3)	**1.48 (1.17–1.88)**	1.21 (0.93–1.58)	71 (9.32)	164 (7.37)	1.29 (0.96–1.73)	1.20 (0.89–1.63)

CAD, Coronary Artery Disease; CI, Confidence Interval; CKD, Chronic Kidney Disease; OR, Odds Ratio; PVD, Peripheral Vascular Disease; VHD, Valvular Heart Disease.

^a^Baseline demographics with a *P*-value ≤.05 were considered significantly different among groups. Adjustments were made for: diabetes, age, gender, obesity, smoking, hypertension, dyslipidaemia, peripheral vascular disease, valvular heart disease, chronic kidney disease, coronary artery disease, and cerebrovascular disease.

^b^The propensity scores were matched in a 1:3 ratio using exact matching on: age (categorical), gender, smoking, hypertension, dyslipidaemia, and CAD.

### 1-year secondary outcomes

**Figure 2:**
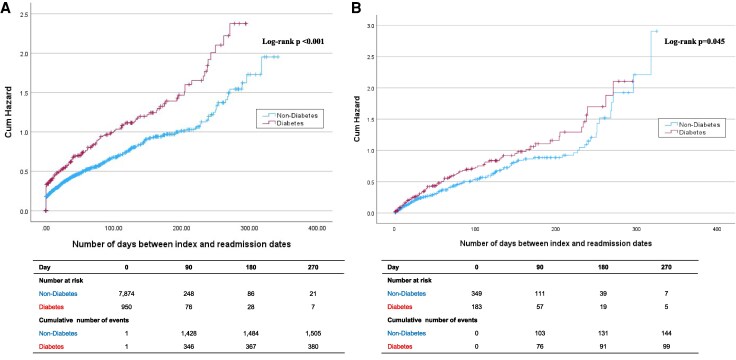
Kaplan–Meier curves for 1-year readmission for heart failure in the (*A*) overall and (*B*) matched patients with myocarditis

We did not observe any statistically significant difference between the survival curves of diabetes and non-diabetes groups for 1-year mortality [HR = 1.19 (95% CI: 0.82–1.17)] (Supplementary Figure S1A) or all-cause readmission [HR = 0.89 (95% CI: 0.77–1.03)] (Supplementary Figure S1B). These results remained unchanged after adjusting for variables that differed between the two groups [aHR= 0.81 (95% CI: 0.41–1.60) and aHR = 0.81 (95% CI: 0.68–0.97) for mortality and readmission, respectively]. However, the 1-year risk of readmission for heart failure was higher by almost 13% in patients with diabetes [HR = 1.13 (95% CI: 1.01–1.27) (*Figure 2A*) and remained significantly higher after adjustment [aHR = 1.16 (95% CI: 1.02–1.31)].

Readmitted patients with diabetes were, on average, 10 years older than those without diabetes (58[16] vs. 48[19], respectively, *P* < .001) (*Table 3*). The proportion of males was higher than that of females in all patients. Cardiometabolic risk factors and previous CVD were significantly higher in patients with diabetes, except for smoking and VHD. Unspecified myocarditis was the most common discharge aetiology in all readmitted patients. However, those with infective myocarditis were more likely to have diabetes (33.5% vs 26.6%, diabetes vs non-diabetes, *P* = .037).

**Table 3:** Comparison of baseline demographics between readmitted myocarditis patients

		Diabetes *n* = 233	Non-diabetes *n* = 870	*P*-value^a^
		*n* (%)	*n* (%)	
Age	Mean (SD)	58 (16)	48 (19)	<.001
	<55	92 (39.5)	542 (62.3)	<.001
	55–64	51 (21.9)	130 (14.9)	
	65–74	57 (24.5)	115 (13.2)	
	75–84	28 (12.0)	63 (7.2)	
	>84	5 (2.2)	20 (2.3)	
Gender	Male	130 (55.8)	453 (52.1)	.312
	Female	103 (44.2)	417 (47.9)	
Comorbidities	Obesity	80 (34.3)	135 (15.5)	<.001
	Smoking	49 (21.0)	166 (19.1)	.505
	Hypertension	189 (81.1)	379 (43.6)	<.001
	Dyslipidaemia	117 (50.2)	221 (25.4)	<.001
	PVD	12 (5.2)	36 (4.1)	.501
	VHD	40 (17.17)	127 (14.6)	.331
	CKD	63 (27.0)	79 (9.1)	<.001
	CAD	90 (38.6)	223 (25.6)	<.001
	Cerebrovascular Disease	19 (8.2)	40 (4.6)	.032
Etiology	Infective acute myocarditis^b^	78 (33.5)	231 (26.6)	.037
	Isolated acute myocarditis^c^	15 (6.4)	43 (4.9)	.364
	Other and unspecified acute myocarditis^d^	40 (17.2)	181 (20.8)	.218
	Unspecified myocarditis^e^	100 (42.9)	415 (47.7)	.194

CAD, Coronary Artery Disease; CKD, Chronic Kidney Disease; PVD, Peripheral Vascular Disease; VHD, Valvular Heart Disease.

^a^Covariates with a *P*-value ≤.05 were considered statistically significant.

^b^Myocarditis caused by a specific infectious agent, such as a virus or bacterium.

^c^Acute myocarditis occurring without an apparent infection or autoimmune disease.

^d^Acute myocarditis that does not fit into the defined categories but has a known cause.

^e^Myocarditis with no specified cause, or additional classification details.

Similarly, readmitted diabetes patients for heart failure were older than their non-diabetes counterparts (*Table 4*). A higher percentage of male patients is evident in all patients. Patients with diabetes had a higher prevalence of risk factors, except for smoking. In terms of previous cardiovascular events, the proportion of patients with CKD was almost twice in the presence of diabetes. Interestingly, infective myocarditis was the most common discharge aetiology in patients readmitted for HF. However, there was no statistical difference in its prevalence according to diabetes, nor in any of the other aetiologies.

**Table 4:** Comparison of baseline demographics of myocarditis patients readmitted for heart failure

		Diabetes *n* = 126	Non-diabetes *n* = 311	*P*-value^a^
		*n* (%)	*n* (%)	
Age	Mean (SD)	59 (15)	51 (18)	<.001
	<55	47 (37.3)	174 (56.0)	.003
	55–64	25 (19.8)	56 (18.0)	
	65–74	35 (27.8)	47 (15.1)	
	75–84	16 (12.7)	25 (8.0)	
	>84	3 (2.4)	9 (2.9)	
Gender	Male	70 (55.6)	166 (53.4)	.679
	Female	56 (44.4)	145 (46.6)	
Comorbidities	Obesity	45 (35.7)	52 (16.7)	<.001
	Smoking	30 (23.8)	62 (19.9)	.368
	Hypertension	110 (87.3)	169 (54.3)	<.001
	Dyslipidaemia	60 (47.6)	80 (25.7)	<.001
	PVD	8 (6.4)	13 (4.2)	.337
	VHD	28 (22.2)	72 (23.2)	.834
	CKD	46 (36.5)	47 (15.1)	<.001
	CAD	50 (39.7)	96 (30.9)	.077
	Cerebrovascular disease	10 (7.9)	16 (5.1)	.264
Etiology	Infective acute myocarditis^b^	50 (39.7)	116 (37.3)	.642
	Isolated acute myocarditis^c^	12 (9.5)	23 (7.4)	.458
	Other and unspecified acute myocarditis^d^	24 (19.1)	71 (22.8)	.385
	Unspecified myocarditis^e^	40 (31.8)	101 (32.5)	.882

CAD, Coronary Artery Disease; CKD, Chronic Kidney Disease; PVD, Peripheral Vascular Disease; VHD, Valvular Heart Disease.

^a^Covariates with a *P*-value ≤.05 were considered statistically significant.

^b^Myocarditis caused by a specific infectious agent, such as a virus or bacterium.

^c^Acute myocarditis occurring without an apparent infection or autoimmune disease.

^d^Acute myocarditis that does not fit into the defined categories but has a known cause.

^e^Myocarditis with no specified cause, or additional classification details.

Supplementary Table S4 shows the Cox Proportional Hazards models for outcomes of interest among patients with and without diabetes. As expected, age was associated with a higher risk of mortality, all-cause readmission, and readmission for heart failure in all patients. Among non-diabetes patients, females had a higher risk of being readmitted for heart failure compared to men [HR = 1.21 (95% CI: 1.09–1.34)]. VHD, CKD, and CAD increased the risk of heart failure readmission in diabetes patients.

### Sensitivity analysis:

Propensity score matching was performed in a 1:3 ratio to compare patients with and without diabetes as a sensitivity analysis. Out of the 951 patients with diabetes in the original cohort, 762 (80.1%) were successfully matched. After matching, the final matched sample included a total of 2986 patients: 762 with diabetes (25.5%) and 2224 without diabetes (74.5%). Additional descriptives are available in Supplementary Tables S5–S7. Comparisons between groups were adjusted for covariates that were significant in the primary analysis. After matching and adjustment, the associations with ARF [Propensity score adjusted OR [AOR] = 1.70 (95% CI: 1.35–2.13)], cardiogenic shock [AOR = 1.43 (95% CI: 1.06–1.94)], and heart failure [AOR = 1.71 (95% CI: 1.42–2.06)] remained significant (*Table 2*). A total of 532 matched patients were readmitted, 184 (34.4%) with diabetes and 349 (65.6%) without diabetes. The 1-year risk of readmission for heart failure among matched patients also remained significant [AHR = 1.13 (95% CI: 1.02–1.46) (*Figure 2B*).

## Discussion

To the best of our knowledge, we are the first to assess the impact of diabetes on myocarditis and show that those patients present a higher risk of heart failure. However, in young type 1 diabetes patients, there are case reports of myocarditis secondary to diabetic ketoacidosis^21–24^ or immune checkpoint inhibitors for metastatic cancer.^25^

Diabetic heart failure shares the same pathophysiological changes as other diabetes complications, such as the accumulation of advanced glycation end products, oxidative stress, low-grade inflammation, disruption of intracellular calcium handling, and capillary damage .^26,27^ As a consequence of these mechanisms, structural changes in the heart are observed, such as interstitial and perivascular fibrosis, myocyte hypertrophy, lipid droplet accumulation, glycogen accumulation in the myocardium, and autophagy.^28^ Echocardiographic findings often reveal myocardial hypertrophy and impaired relaxation despite a normal ejection fraction,^18^ although the latter frequently decreases over time.^29^ The presence of myocarditis in patients with diabetes may be associated with greater inflammation, worsened myocardial injury, and exacerbated maladaptive remodelling, potentially increasing the risk of heart failure.^30,31^ It is also possible that patients with myocarditis and diabetes experience delayed healing and fibrosis, resulting in ventricular stiffening and systolic dysfunction. Delayed healing could result from hyperglycaemia-induced dysfunction of fibroblasts and the deposition of extracellular matrix proteins.^32^ These mechanisms remain speculative in the context of our findings and warrant further study.

We acknowledge the limitations of this study. The NRD is an administrative dataset that lacks several important parameters, including medication information, HbA1c levels, diabetes type, and duration. Further, diagnoses, comorbidities, and clinical events were coded using ICD-10, which may introduce misclassification and selection bias. Furthermore, the criteria and parameters used to define obesity, dyslipidaemia, and other risk factors are not available in the NRD database. The absence of competing-risk modelling for readmission and the lack of sampling weights may slightly limit the accuracy of our estimates and the generalizability of the findings. Furthermore, a causality between diabetes and the excess heart failure risk in patients with myocarditis could not be established in the analysis of data from the NRD. Despite adjusting for baseline comorbidities, residual confounding cannot be ruled out due to the observational nature of our study. As such, our findings should be interpreted as associative rather than causal. Since our outcome was common, the reported odds ratios may slightly overestimate the magnitude of associations compared with risk ratios.^33^ The total number of deaths during the follow-up was small, making it difficult to comment on those results conclusively. A higher incidence or a larger number of patients could have resulted in a significant difference between the two groups. We did not account for the competing risk of mortality in the analysis of readmission using Fine–Gray models. This may have led to a slight overestimation of readmission risk and should be taken into consideration in future studies. The observed reduction in 1-year mortality among patients with diabetes should be interpreted cautiously, as it may be influenced by residual confounding, variations in post-discharge care, or competing clinical events. The dataset also lacked information on patient race or ethnicity, limiting our ability to explore potential disparities or trends across populations. Since the NRD includes only inpatient data, our analysis is limited to hospitalized myocarditis cases, which may introduce selection bias by excluding milder, outpatient-managed presentations. Deaths occurring in the community were not captured or analysed. The NRD does not provide data on medication use during hospitalization, which limits the ability to adjust for therapies. Sampling weights were unavailable and were therefore not applied; results represent unweighted estimates from the NRD dataset. Future studies may consider applying national weights to improve the generalizability of the results.

However, we believe that we were able to establish a negative impact of diabetes on myocarditis, reflected by a higher incidence of heart failure. One of the strengths of our study is the analysis of NRD data, one of the largest clinical databases in the United States, which is ideal for assessing the course of events in uncommon pathologies, such as diabetes in patients with myocarditis. Additionally, the NRD provides nationally representative information on hospital readmissions for all ages. Furthermore, as a sensitivity analysis, we used a doubly robust statistical approach by combining propensity score matching with outcome regression. This helps protect against model misspecification in either the propensity score model or the outcome model.

Our findings identify diabetes as a key risk modifier in patients with myocarditis, with direct implications for post-discharge management. However, the magnitude of this association may vary according to the underlying aetiology of myocarditis (e.g. viral, autoimmune, or toxic), and further studies are needed to determine whether specific causes modify the impact of diabetes on heart failure risk. Diabetes patients may warrant intensified surveillance and earlier reassessment of ventricular function, even when the ejection fraction is initially preserved. Incorporating diabetes status into risk stratification frameworks could facilitate earlier detection of adverse remodelling and progression to heart failure, thereby improving outcomes. Future clinical studies should focus on interventions to reduce excess cardiovascular risk in these patients, such as tight in-hospital glycaemic control and the preventive use of medications known to reduce cardiovascular risk, such as SGLT-2 inhibitors.

## Supplementary Material

xvag064_Supplementary_Data

## Declarations

### Data Availability

Data are available upon reasonable request from the lead author.
