# Supplementary material for: Diabetes increases the risk of heart failure in myocarditis: a propensity-matched nationwide database analysis
Source: ESC Heart Fail. 2026 Feb 23;13(3):xvag064. doi: 10.1093/eschf/xvag064 (PMC13244792; doi:10.1093/eschf/xvag064)
Supplement: xvag064_Supplementary_Data [file xvag064_supplementary_data.zip › Supplementary Figure 1_revised.pptx]

## Slide 1
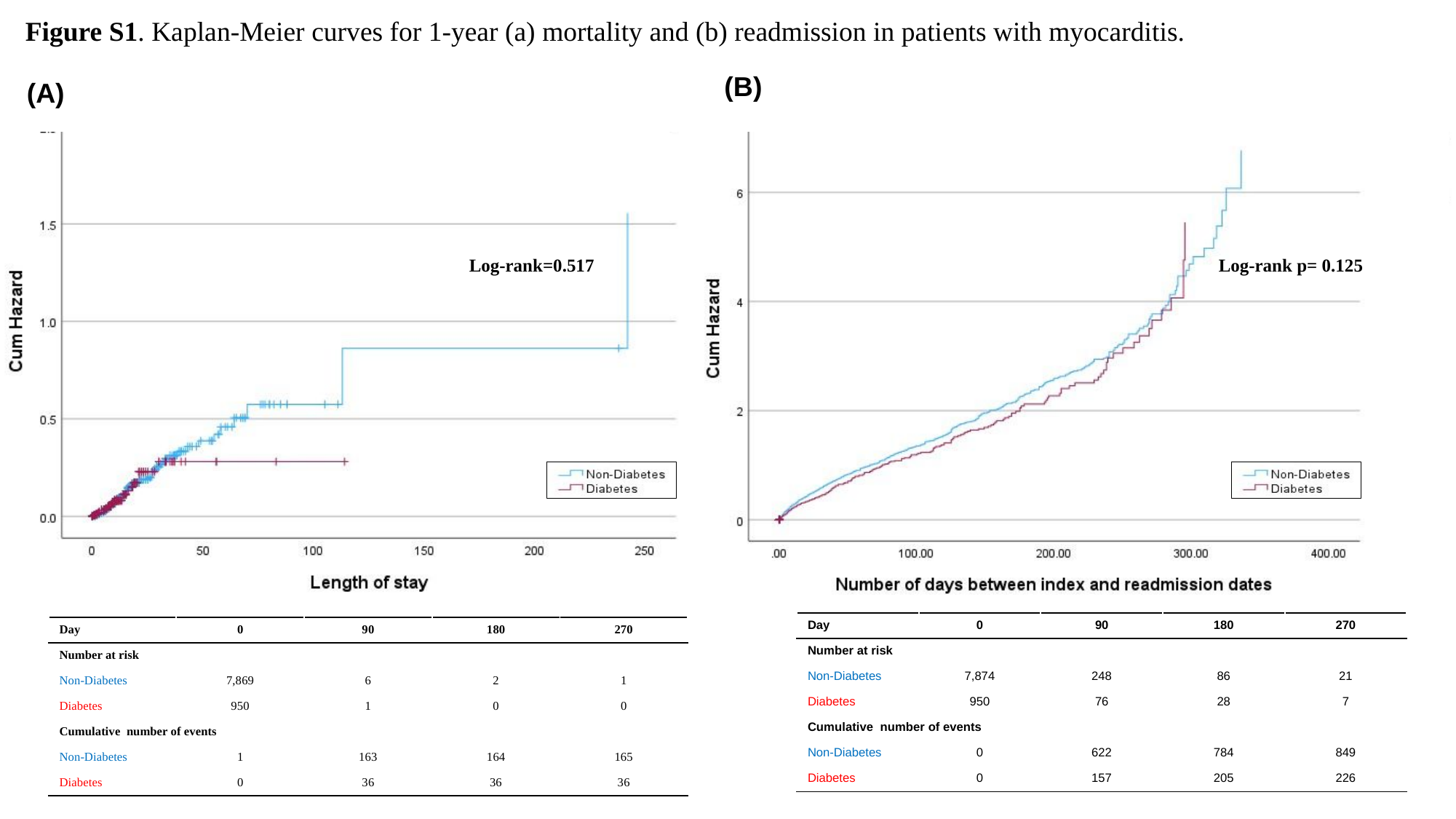

Figure S1. Kaplan-Meier curves for 1-year (a) mortality and (b) readmission in patients with myocarditis.
(B)
(A)
Log-rank=0.517
Log-rank p= 0.125
| Day | 0 | 90 | 180 | 270 |
| --- | --- | --- | --- | --- |
| Number at risk | | | | |
| Non-Diabetes | 7,874 | 248 | 86 | 21 |
| Diabetes | 950 | 76 | 28 | 7 |
| Cumulative number of events | | | | |
| Non-Diabetes | 0 | 622 | 784 | 849 |
| Diabetes | 0 | 157 | 205 | 226 |
| Day | 0 | 90 | 180 | 270 |
| --- | --- | --- | --- | --- |
| Number at risk | | | | |
| Non-Diabetes | 7,869 | 6 | 2 | 1 |
| Diabetes | 950 | 1 | 0 | 0 |
| Cumulative number of events | | | | |
| Non-Diabetes | 1 | 163 | 164 | 165 |
| Diabetes | 0 | 36 | 36 | 36 |
