# Supplementary material for: Diabetes increases the risk of heart failure in myocarditis: a propensity-matched nationwide database analysis
Source: ESC Heart Fail. 2026 Feb 23;13(3):xvag064. doi: 10.1093/eschf/xvag064 (PMC13244792; doi:10.1093/eschf/xvag064)
Supplement: xvag064_Supplementary_Data [file xvag064_supplementary_data.zip › Supplementary Tables_revised_clean.docx]

**Table S1.** STROBE Statement—Checklist of items that should be included in reports of ***cross-sectional studies***.

|  | Item No | Recommendation | Page No |
| --- | --- | --- | --- |
| **Title and abstract** | 1 | (*a*) Indicate the study’s design with a commonly used term in the title or the abstract | **Pg.1** |
|  |  | (*b*) Provide in the abstract an informative and balanced summary of what was done and what was found | **Pg.2** |
| Introduction | | | |
| Background/rationale | 2 | Explain the scientific background and rationale for the investigation being reported | **Pg.3** |
| Objectives | 3 | State specific objectives, including any prespecified hypotheses | **Pg.3** |
| Methods | | | |
| Study design | 4 | Present key elements of study design early in the paper | **Pg.3** |
| Setting | 5 | Describe the setting, locations, and relevant dates, including periods of recruitment, exposure, follow-up, and data collection | **Pg.3,4** |
| Participants | 6 | (*a*) Give the eligibility criteria, and the sources and methods of selection of participants | **Pg.4** |
| Variables | 7 | Clearly define all outcomes, exposures, predictors, potential confounders, and effect modifiers. Give diagnostic criteria, if applicable | **Pg.4** |
| Data sources/ measurement | 8* | For each variable of interest, give sources of data and details of methods of assessment (measurement). Describe comparability of assessment methods if there is more than one group | **Pg.4** |
| Bias | 9 | Describe any efforts to address potential sources of bias | **NA** |
| Study size | 10 | Explain how the study size was arrived at | **Pg.3,4** |
| Quantitative variables | 11 | Explain how quantitative variables were handled in the analyses. If applicable, describe which groupings were chosen and why | **Pg.4,5** |
| Statistical methods | 12 | (*a*) Describe all statistical methods, including those used to control for confounding | **Pg.4,5** |
|  |  | (*b*) Describe any methods used to examine subgroups and interactions | **NA** |
|  |  | (*c*) Explain how missing data were addressed | **NA** |
|  |  | (*d*) If applicable, describe analytical methods taking account of sampling strategy | **NA** |
|  |  | (*e*) Describe any sensitivity analyses | **Pg.5** |
| Results | | | |
| Participants | 13* | (a) Report numbers of individuals at each stage of study—eg numbers potentially eligible, examined for eligibility, confirmed eligible, included in the study, completing follow-up, and analysed | **Pg.5, Fig.1** |
|  |  | (b) Give reasons for non-participation at each stage | **Pg.5** |
|  |  | (c) Consider use of a flow diagram | **Pg.5, Fig.1** |
| Descriptive data | 14* | (a) Give characteristics of study participants (eg demographic, clinical, social) and information on exposures and potential confounders | **Pg.5-7** |
|  |  | (b) Indicate number of participants with missing data for each variable of interest | **Pg.5-7** |
| Outcome data | 15* | Report numbers of outcome events or summary measures | **Pg.5-7** |
| Main results | 16 | (*a*) Give unadjusted estimates and, if applicable, confounder-adjusted estimates and their precision (eg, 95% confidence interval). Make clear which confounders were adjusted for and why they were included | **Pg.5-7, Table 2** |
|  |  | (*b*) Report category boundaries when continuous variables were categorized | **Pg.5-7** |
|  |  | (*c*) If relevant, consider translating estimates of relative risk into absolute risk for a meaningful time period | **Pg.5-7, Table S3** |
| Other analyses | 17 | Report other analyses done—eg analyses of subgroups and interactions, and sensitivity analyses | **Pg.7,8** |
| Discussion | | | |
| Key results | 18 | Summarise key results with reference to study objectives | **Pg.8** |
| Limitations | 19 | Discuss limitations of the study, taking into account sources of potential bias or imprecision. Discuss both direction and magnitude of any potential bias | **Pg.9,10** |
| Interpretation | 20 | Give a cautious overall interpretation of results considering objectives, limitations, multiplicity of analyses, results from similar studies, and other relevant evidence | **Pg.10** |
| Generalisability | 21 | Discuss the generalisability (external validity) of the study results | **Pg.10** |
| Other information | | | |
| Funding | 22 | Give the source of funding and the role of the funders for the present study and, if applicable, for the original study on which the present article is based | **Pg.11** |

*Give information separately for exposed and unexposed groups.

**Note:** An Explanation and Elaboration article discusses each checklist item and gives methodological background and published examples of transparent reporting. The STROBE checklist is best used in conjunction with this article (freely available on the Web sites of PLoS Medicine at http://www.plosmedicine.org/, Annals of Internal Medicine at http://www.annals.org/, and Epidemiology at http://www.epidem.com/). Information on the STROBE Initiative is available at www.strobe-statement.org.

**Table S2.** The ICD-10 codes for study variables.

| **Variables** | **ICD-10 codes used** |
| --- | --- |
| **Obesity** | E6601, E6609, E661, E662, E668, E669, O99210, O99211, O99212, O99213, O99214, O99215, R939, Z6830, Z6831, Z6832, Z6833, Z6834, Z6835, Z6836, Z6837, Z6838, Z6839, Z6841, Z6842, Z6843, Z6844, Z6845, Z6854. |
| **Smoking** | Z87891, F1720, F1721. |
| **Hypertension** | E000, E001, E002, E009, E018, E02, E030, E031, E032, E033, E038, E039, E890. |
| **Dyslipidemia** | E780, E781, E782, E783, E784, E785. |
| **Peripheral Vascular Disease** | F200, F201, F202, F203, F205, F2081, F2089, F209, F22, F23, F24, F250, F251, F258, F259, F28, F29, F3010, F3011, F3012, F3013, F302, F303, F304, F308, F309, F310, F3110, F3111, F3112, F3113, F312, F3130, F3131, F3132, F314, F315, F3160, F3161, F3162, F3163, F3164, F3170, F3171, F3172, F3173, F3174, F3175, F3176, F3177, F3178, F3181, F3189, F319, F324, F325, F3340, F3341, F3342, F348, F349, F39, F4489, F843. |
| **Valvular Heart Disease** | A5203, I050, I051, I052, I058, I059, I060, I061, I062, I068, I069, I070, I071, I072, I078, I079, I080, I081, I082, I083, I088, I089, I091, I0989, I340, I341, I342, I348, I349, I350, I351, I352, I358, I359, I360, I361, I362, I368, I369, I370, I371, I372, I378, I379, I38, I39, Q230, Q231, Q232, Q233, Z952, Z953, Z954. |
| **Chronic Kidney Disease** | N183, N184, N185, N186, N189, N19, Z4901, Z4902, Z9115, Z940, Z992, Z4931, Z4932. |
| **Coronary Artery Disease** | I2510, I2511, I252, I2582, I2584, Z955, Z951. |
| **Cerebrovascular Disease** | Z8673. |
| **Etiology** | - Infective acute myocarditis: I400. - Isolated acute myocarditis: I401. - Other and unspecified acute myocarditis: I408, I409. - Unspecified myocarditis: I514. |
| **Acute Renal Failure** | N170, N171, N172, N178, N179. |
| **Cardiogenic Shock** | R570. |
| **Heart Failure** | I110, I130, I132, I509, I5030, I5031, I5032, I5033, I50814, I1501, I15020, I15021, I15022, I15023, I15040, I15041, I15042, I15043, I150810, I150811, I150812, I150813, I15082, I15083, I15084, I159089. |

**Table S3.** Predictors of in-hospital outcomes among myocarditis patients.

|  |  | **ARF** | | | | **Cardiogenic Shock** | | | | **Heart failure** | | | |
| --- | --- | --- | --- | --- | --- | --- | --- | --- | --- | --- | --- | --- | --- |
|  |  | **Diabetes** | | **Non-diabetes** | | **Diabetes** | | **Non-diabetes** | | **Diabetes** | | **Non-diabetes** | |
|  |  | **OR (95% CI)** | **p-value** | **OR (95% CI)** | **p-value** | **OR (95% CI)** | **p-value** | **OR (95% CI)** | **p-value** | **OR (95% CI)** | **p-value** | **OR (95% CI)** | **p-value** |
| **Age** |  | 1.01 (1.00-1.02) | 0.021 | 1.03 (1.02-1.03) | <0.001 | 1.00 (0.98-1.02) | 0.976 | 1.02 (1.01-1.02) | <0.001 | 1.01 (1.00-1.02) | 0.004 | 1.02 (1.01-1.02) | <0.001 |
| **Gender** | **Male** | Ref | - | Ref | - | Ref | - | Ref | - | Ref | - | Ref | - |
|  | **Female** | 0.64 (0.46-0.88) | 0.006 | 0.66 (0.56-0.79) | <0.001 | 0.80 (0.50-1.26) | 0.329 | 1.35 (1.10-1.66) | 0.005 | 0.89 (0.67-1.17) | 0.394 | 1.06 (0.93-1.20) | 0.384 |
| **Obesity** | **No** | Ref | - | Ref | - | Ref | - | Ref | - | Ref | - | Ref | - |
|  | **Yes** | 0.99 (0.70-1.39) | 0.931 | 1.18 (0.95-1.47) | 0.135 | 0.83 (0.50-1.35) | 0.446 | 0.92 (0.69-1.23) | 0.592 | 1.12 (0.84-1.51) | 0.436 | 1.33 (1.14-1.57) | <0.001 |
| **Smoking** | **No** | Ref | - | Ref | - | Ref | - | Ref | - | Ref | - | Ref | - |
|  | **Yes** | 0.78 (0.52-1.17) | 0.224 | 0.77 (0.61-0.97) | 0.027 | 0.69 (0.37-1.27) | 0.233 | 0.72 (0.53-0.96) | 0.028 | 1.04 (0.74-1.47) | 0.815 | 0.96 (0.81-1.13) | 0.600 |
| **Hypertension** | **No** | Ref | - | Ref | - | Ref | - | Ref | - | Ref | - | Ref | - |
|  | **Yes** | 0.92 (0.61-1.37) | 0.671 | 1.32 (1.10-1.60) | 0.004 | 0.92 (0.53-1.60) | 0.780 | 1.04 (0.82-1.32) | 0.746 | 1.07 (0.75-1.51) | 0.721 | 1.78 (1.55-2.05) | <0.001 |
| **Dyslipidemia** | **No** | Ref | - | Ref | - | Ref | - | Ref | - | Ref | - | Ref | - |
|  | **Yes** | 0.62 (0.45-0.87) | 0.005 | 0.61 (0.49-0.75) | <0.001 | 0.62 (0.38-0.98) | 0.042 | 0.52 (0.39-0.69) | <0.001 | 0.56 (0.42-0.75) | <0.001 | 0.62 (0.3-0.73) | <0.001 |
| **PVD** | **No** | Ref | - | Ref | - | Ref | - | Ref | - | Ref | - | Ref | - |
|  | **Yes** | 1.10 (0.56-2.15) | 0.784 | 2.94 (2.05-4.21) | <0.001 | 1.32 (0.52-3.35) | 0.563 | 3.10 (2.06-4.66) | <0.001 | 0.91 (0.49-1.70) | 0.772 | 1.33 (0.95-1.86) | 0.097 |
| **VHD** | **No** | Ref | - | Ref | - | Ref | - | Ref | - | Ref | - | Ref | - |
|  | **Yes** | 1.30 (0.84-2.03) | 0.240 | 1.75 (1.38-2.22) | <0.001 | 2.02 (1.16-3.53) | 0.013 | 2.31 (1.77-3.01) | <0.001 | 2.30 (1.55-3.42) | <0.001 | 3.41 (2.86-4.07) | <0.001 |
| **CKD** | **No** | Ref | - | Ref | - | Ref | - | Ref | - | Ref | - | Ref | - |
|  | **Yes** | 4.13 (2.84-6.01) | <0.001 | 8.56 (6.51-11.27) | <0.001 | 1.19 (0.67-2.12) | 0.558 | 3.10 (2.18-4.40) | <0.001 | 2.21 (1.54-3.17) | <0.001 | 3.23 (2.48-4.20) | <0.001 |
| **CAD** | **No** | Ref | - | Ref | - | Ref | - | Ref | - | Ref | - | Ref | - |
|  | **Yes** | 1.04 (0.74-1.46) | 0.823 | 0.67 (0.53-0.84) | 0.001 | 1.24 (0.76-2.01) | 0.388 | 0.86 (0.65-1.13) | 0.278 | 1.22 (0.91-1.64) | 0.183 | 1.07 (0.91-1.27) | 0.401 |
| **Cerebrovascular Disease** | **No** | Ref | - | Ref | - | Ref | - | Ref | - | Ref | - | Ref | - |
|  | **Yes** | 1.09 (0.57-2.11) | 0.787 | 0.76 (0.44-1.32) | 0.334 | 0.35 (0.08-1.48) | 0.154 | 0.28 (0.10-0.78) | 0.015 | 1.08 (0.59-1.96) | 0.800 | 0.98 (0.66-1.46) | 0.927 |

Abbreviations: ARF = Acute Renal Failure; CAD = Coronary Artery Disease; CI = Confidence Interval, CKD = Chronic Kidney Disease; OR = Odds Ratio; PVD = Peripheral Vascular Disease; VHD = Valvular Heart Disease.

**Table S4.** Cox Proportional Hazards models for outcomes of interest among myocarditis patients.

|  |  | **All-cause readmission** | | | | **Mortality** | | | | **Heart failure** | | | |
| --- | --- | --- | --- | --- | --- | --- | --- | --- | --- | --- | --- | --- | --- |
|  |  | **Diabetes** | | **Non-diabetes** | | **Diabetes** | | **Non-diabetes** | | **Diabetes** | | **Non-diabetes** | |
|  |  | **HR (95% CI)** | **p-value** | **HR (95% CI)** | **p-value** | **HR (95% CI)** | **p-value** | **HR (95% CI)** | **p-value** | **HR (95% CI)** | **p-value** | **HR (95% CI)** | **p-value** |
| **Age** |  | 1.00 (0.99-1.01) | 0.746 | 1.00 (1.00-1.01) | 0.139 | 1.03 (1.01-1.05) | 0.009 | 1.04 (1.03-1.05) | <0.001 | 1.01 (1.00-1.02) | 0.015 | 1.02 (1.01-1.02) | <0.001 |
| **Gender** | **Male** | Ref | - | Ref | - | Ref | - | Ref | - | Ref | - | Ref | - |
|  | **Female** | 1.00 (0.77-1.30) | 0.969 | 1.01 (0.88-1.16) | 0.879 | 1.09 (0.57-2.10) | 0.798 | 1.34 (0.99-1.83) | 0.061 | 0.98 (0.80-1.20) | 0.821 | 1.21 (1.09-1.34) | <0.001 |
| **Obesity** | **No** | Ref | - | Ref | - | Ref | - | Ref | - | Ref | - | Ref | - |
|  | **Yes** | 1.00 (0.76-1.31) | 0.979 | 0.91 (0.76-1.10) | 0.342 | 0.90 (0.43-1.88) | 0.783 | 0.70 (0.42-1.15) | 0.158 | 0.96 (0.78-1.19) | 0.704 | 1.26 (1.11-1.44) | <0.001 |
| **Smoking** | **No** | Ref | - | Ref | - | Ref | - | Ref | - | Ref | - | Ref | - |
|  | **Yes** | 1.10 (0.79-1.49) | 0.610 | 0.95 (0.80-1.12) | 0.540 | 0.56 (0.20-1.59) | 0.276 | 1.16 (0.75-1.78) | 0.502 | 1.12 (0.89-1.44) | 0.328 | 1.11 (0.97-1.27) | 0.120 |
| **Hypertension** | **No** | Ref | - | Ref | - | Ref | - | Ref | - | Ref | - | Ref | - |
|  | **Yes** | 1.09 (0.79-1.52) | 0.591 | 0.96 (0.84-1.09) | 0.507 | 2.03 (0.79-5.24) | 0.142 | 1.35 (0.99-1.83) | 0.060 | 1.10 (0.86-1.41) | 0.465 | 1.83 (1.66-2.03) | <0.001 |
| **Dyslipidemia** | **No** | Ref | - | Ref | - | Ref | - | Ref | - | Ref | - | Ref | - |
|  | **Yes** | 0.89 (0.69-1.15) | 0.373 | 1.04 (0.90-1.22) | 0.591 | 1.28 (0.66-2.48) | 0.467 | 1.38 (0.95-1.99) | 0.087 | 0.85 (0.70-1.04) | 0.122 | 1.08 (0.96-1.22) | 0.197 |
| **PVD** | **No** | Ref | - | Ref | - | Ref | - | Ref | - | Ref | - | Ref | - |
|  | **Yes** | 1.27 (0.71-2.27) | 0.428 | 1.38 (0.99-1.92) | 0.060 | 0.42 (0.58-3.09) | 0.397 | 1.65 (0.97-2.81) | 0.065 | 1.28 (0.84-1.95) | 0.257 | 1.71 (1.33-2.19) | <0.001 |
| **VHD** | **No** | Ref | - | Ref | - | Ref | - | Ref | - | Ref | - | Ref | - |
|  | **Yes** | 0.97 (0.69-1.36) | 0.843 | 1.10 (0.91-1.33) | 0.323 | 0.91 (0.38-2.19) | 0.828 | 1.17 (0.76-1.80) | 0.480 | 1.42 (1.10-1.83) | 0.006 | 2.36 (2.08-2.69) | <0.001 |
| **CKD** | **No** | Ref | - | Ref | - | Ref | - | Ref | - | Ref | - | Ref | - |
|  | **Yes** | 0.97 (0.72-1.30) | 0.822 | 1.00 (0.80-1.26) | 0.984 | 1.07 (0.51-2.24) | 0.862 | 1.85 (1.23-2.78) | 0.003 | 1.32 (1.05-1.66) | 0.017 | 2.34 (1.98-2.77) | <0.001 |
| **CAD** | **No** | Ref | - | Ref | - | Ref | - | Ref | - | Ref | - | Ref | - |
|  | **Yes** | 1.01 (0.77-1.32) | 0.943 | 0.98 (0.84-1.14) | 0.806 | 0.87 (0.44-1.75) | 0.699 | 1.67 (1.17-2.37) | 0.004 | 1.17 (0.96-1.44) | 0.127 | 1.35 (1.19-1.52) | <0.001 |
| **Cerebrovascular Disease** | **No** | Ref | - | Ref | - | Ref | - | Ref | - | Ref | - | Ref | - |
|  | **Yes** | 0.75 (0.47-1.19) | 0.221 | 1.03 (0.75-1.42) | 0.852 | 0.67 (0.91-4.93) | 0.694 | 0.91 (0.29-2.86) | 0.876 | 0.87 (0.58-1.32) | 0.514 | 1.24 (0.91-1.68) | 0.169 |

Abbreviations: CAD = Coronary Artery Disease; CI = Confidence Interval; CKD = Chronic Kidney Disease; HR = Hazard Ratio; PVD = Peripheral Vascular Disease; VHD = Valvular Heart Disease.

**Table S5.** Comparison of baseline demographics between readmitted myocarditis patients among the matched population.

|  |  | Diabetes  n= 183 | Non-diabetes  n= 349 |  |
| --- | --- | --- | --- | --- |
|  |  | **n (%)** | **n (%)** | **p-value^*^** |
| Age | Mean (SD) | 55 (16) | 58 (17) | 0.198 |
|  | <55 | 86 (47.0) | 129 (37.0) | 0.194 |
|  | 55 - 64 | 42 (23.0) | 84 (24.1) |  |
|  | 65 - 74 | 36 (19.7) | 83 (23.8) |  |
|  | 75 - 84 | 16 (8.7) | 46 (13.2) |  |
|  | >84 | 3 (1.6) | 7 (2.0) |  |
| Gender | Male | 101 (55.2) | 177 (50.7) | 0.326 |
|  | Female | 82 (44.8) | 172 (49.3) |  |
| Comorbidities | Obesity | 67 (36.6) | 59 (16.9) | <0.001 |
|  | Smoking | 37 (20.2) | 77 (22.1) | 0.622 |
|  | Hypertension | 141 (77.1) | 275 (78.8) | 0.643 |
|  | Dyslipidemia | 74 (40.4) | 166 (47.6) | 0.117 |
|  | PVD | 9 (4.9) | 22 (6.3) | 0.517 |
|  | VHD | 27 (14.8) | 62 (17.8) | 0.377 |
|  | CKD | 48 (26.2) | 51 (14.6) | 0.001 |
|  | CAD | 62 (33.9) | 135 (38.7) | 0.276 |
|  | Cerebrovascular Disease | 12 (6.6) | 21 (6.0) | 0.806 |
| Etiology | Infective acute myocarditis^†^ | 60 (32.8) | 96 (27.5) | 0.204 |
|  | Isolated acute myocarditis^‡^ | 11 (6.0) | 20 (5.7) | 0.896 |
|  | Other and unspecified acute myocarditis^¶^ | 32 (17.5) | 72 (20.6) | 0.385 |
|  | Unspecified myocarditis^§^ | 80 (43.7) | 161 (46.1) | 0.595 |

Abbreviations: CAD = Coronary Artery Disease; CKD = Chronic Kidney Disease; PVD = Peripheral Vascular Disease; VHD = Valvular Heart Disease.

^*^ Covariates with a p-value ≤0.05 were considered statistically significant.

^†^Myocarditis caused by a specific infectious agent, such as a virus or bacterium.

^‡^Acute myocarditis occurring without an apparent infection or autoimmune disease.

^¶^Acute myocarditis that does not fit into the defined categories but has a known cause.

^§^Myocarditis with no specified cause, or additional classification details.

**Table S6.** Comparison of baseline demographics of myocarditis patients readmitted for heart failure among the matched population.

|  |  | Diabetes  n= 101 | Non-diabetes  n= 147 |  |
| --- | --- | --- | --- | --- |
|  |  | **n (%)** | **n (%)** | **p-value^*^** |
| Age | Mean (SD) | 57 (15) | 59 (16) | 0.376 |
|  | <55 | 44 (43.6) | 45 (30.6) | 0.334 |
|  | 55 - 64 | 25 (24.8) | 43 (29.3) |  |
|  | 65 - 74 | 20 (19.8) | 35 (23.8) |  |
|  | 75 - 84 | 10 (9.9) | 19 (12.9) |  |
|  | >84 | 2 (2.0) | 5 (3.4) |  |
| Gender | Male | 56 (55.5) | 81 (55.1) | 0.957 |
|  | Female | 45 (44.6) | 66 (44.9) |  |
| Comorbidities | Obesity | 36 (35.6) | 28 (19.1) | 0.003 |
|  | Smoking | 25 (24.8) | 31 (21.1) | 0.498 |
|  | Hypertension | 85 (84.2) | 123 (83.7) | 0.919 |
|  | Dyslipidemia | 40 (39.6) | 68 (46.3) | 0.299 |
|  | PVD | 5 (5.0) | 7 (4.8) | 0.946 |
|  | VHD | 19 (18.8) | 35 (23.8) | 0.349 |
|  | CKD | 36 (35.6) | 31 (21.1) | 0.011 |
|  | CAD | 34 (33.7) | 61 (41.5) | 0.212 |
|  | Cerebrovascular Disease | 6 (5.9) | 11 (7.5) | 0.637 |
| Etiology | Infective acute myocarditis^†^ | 42 (41.6) | 55 (37.4) | 0.509 |
|  | Isolated acute myocarditis^‡^ | 9 (8.9) | 11 (7.5) | 0.685 |
|  | Other and unspecified acute myocarditis^¶^ | 18 (17.8) | 34 (23.1) | 0.313 |
|  | Unspecified myocarditis^§^ | 32 (31.7) | 47 (32.0) | 0.962 |

Abbreviations: CAD = Coronary Artery Disease; CKD = Chronic Kidney Disease; PVD = Peripheral Vascular Disease; VHD = Valvular Heart Disease.

^*^Covariates with a p-value ≤0.05 were considered statistically significant.

^†^Myocarditis caused by a specific infectious agent, such as a virus or bacterium.

^‡^Acute myocarditis occurring without an apparent infection or autoimmune disease.

^¶^Acute myocarditis that does not fit into the defined categories but has a known cause.

^§^Myocarditis with no specified cause, or additional classification details.

**Table S7.** Cox Proportional Hazards models for outcomes of interest among myocarditis patients among matched patients.

|  |  | **All-cause readmission** | | | | **Mortality** | | | | **Heart failure** | | | |
| --- | --- | --- | --- | --- | --- | --- | --- | --- | --- | --- | --- | --- | --- |
|  |  | **Diabetes** | | **Non-diabetes** | | **Diabetes** | | **Non-diabetes** | | **Diabetes** | | **Non-diabetes** | |
|  |  | **HR (95% CI)** | **p-value** | **HR (95% CI)** | **p-value** | **HR (95% CI)** | **p-value** | **HR (95% CI)** | **p-value** | **HR (95% CI)** | **p-value** | **HR (95% CI)** | **p-value** |
| **Age** |  | 1.00 (0.99-1.01) | 0.962 | 1.01 (1.00-1.01) | 0.013 | 1.03 (1.01-1.06) | 0.015 | 1.03 (1.01-1.06) | 0.015 | 1.01 (0.99-1.02) | 0.236 | 1.02 (1.00-1.03) | 0.004 |
| **Gender** | **Male** | Ref | - | Ref | - | Ref | - | Ref | - | Ref | - | Ref | - |
|  | **Female** | 1.19 (0.89-1.60) | 0.235 | 0.88 (0.71-1.08) | 0.222 | 1.37 (0.66-2.85) | 0.394 | 1.37 (0.66-2.85) | 0.394 | 1.18 (0.90-1.75) | 0.410 | 0.75 (0.54-1.04) | 0.084 |
| **Obesity** | **No** | Ref | - | Ref | - | Ref | - | Ref | - | Ref | - | Ref | - |
|  | **Yes** | 1.05 (0.77-1.42) | 0.764 | 1.11 (0.84-1.47) | 0.458 | 0.97 (0.11-2.13) | 0.937 | 0.97 (0.11-2.13) | 0.937 | 0.99 (0.66-1.51) | 0.989 | 1.29 (0.85-1.95) | 0.225 |
| **Smoking** | **No** | Ref | - | Ref | - | Ref | - | Ref | - | Ref | - | Ref | - |
|  | **Yes** | 1.02 (0.71-1.46) | 0.933 | 0.97 (0.75-1.24) | 0.788 | 0.51 (0.15-1.68) | 0.265 | 0.51 (0.15-1.68) | 0.265 | 1.38 (0.87-2.16) | 0.171 | 0.94 (0.63-1.40) | 0.751 |
| **Hypertension** | **No** | Ref | - | Ref | - | Ref | - | Ref | - | Ref | - | Ref | - |
|  | **Yes** | 1.22 (0.86-1.73) | 0.256 | 0.94 (0.73-1.22) | 0.635 | 2.65 (0.92-7.63) | 0.071 | 2.65 (0.92-7.63) | 0.071 | 1.93 (1.13-3.30) | 0.016 | 1.30 (0.84-2.01) | 0.245 |
| **Dyslipidemia** | **No** | Ref | - | Ref | - | Ref | - | Ref | - | Ref | - | Ref | - |
|  | **Yes** | 0.96 (0.71-1.30) | 0.794 | 0.99 (0.80-1.22) | 0.935 | 1.35 (0.65-2.82) | 0.423 | 1.35 (0.65-2.82) | 0.423 | 0.97 (0.65-1.45) | 0.874 | 0.95 (0.69-1.31) | 0.748 |
| **PVD** | **No** | Ref | - | Ref | - | Ref | - | Ref | - | Ref | - | Ref | - |
|  | **Yes** | 1.22 (0.62-2.40) | 0.558 | 1.30 (0.84-2.00) | 0.237 | 0.55 (0.08-4.08) | 0.561 | 0.55 (0.08-4.08) | 0.561 | 1.20 (0.48-2.95) | 0.699 | 0.97 (0.45-2.08) | 0.939 |
| **VHD** | **No** | Ref | - | Ref | - | Ref | - | Ref | - | Ref | - | Ref | - |
|  | **Yes** | 0.93 (0.62-1.41) | 0.743 | 1.11 (0.84-1.46) | 0.459 | 0.62 (0.19-2.05) | 0.433 | 0.62 (0.19-2.05) | 0.433 | 1.28 (0.77-2.12) | 0.350 | 1.63 (1.11-2.39) | 0.012 |
| **CKD** | **No** | Ref | - | Ref | - | Ref | - | Ref | - | Ref | - | Ref | - |
|  | **Yes** | 0.91 (0.65-1.27) | 0.578 | 1.00 (0.75-1.35) | 0.975 | 0.90 (0.38-2.13) | 0.807 | 0.90 (0.38-2.13) | 0.807 | 1.39 (0.92-2.10) | 0.113 | 1.55 (1.04-2.31) | 0.032 |
| **CAD** | **No** | Ref | - | Ref | - | Ref | - | Ref | - | Ref | - | Ref | - |
|  | **Yes** | 1.04 (0.76-1.41) | 0.817 | 1.01 (0.82-1.26) | 0.900 | 0.79 (0.35-1.79) | 0.572 | 0.79 (0.35-1.79) | 0.572 | 1.04 (0.69-1.57) | 0.868 | 1.14 (0.82-1.58) | 0.450 |
| **Cerebrovascular Disease** | **No** | Ref | - | Ref | - | Ref | - | Ref | - | Ref | - | Ref | - |
|  | **Yes** | 0.93 (0.52-1.67) | 0.809 | 1.03 (0.66-1.60) | 0.896 | 0.99 (0.13-7.43) | 0.998 | 0.99 (0.13-7.43) | 0.998 | 0.83 (0.36-1.89) | 0.651 | 1.28 (0.69-2.36) | 0.436 |

Abbreviations: CAD = Coronary Artery Disease; CI = Confidence Interval; CKD = Chronic Kidney Disease; HR = Hazard Ratio; PVD = Peripheral Vascular Disease; VHD = Valvular Heart Disease.
